# Color reproduction and processing algorithm based on real-time mapping for endoscopic images

**DOI:** 10.1186/s40064-015-1612-4

**Published:** 2016-01-06

**Authors:** Tareq H. Khan, Shahed K. Mohammed, Mohammad S. Imtiaz, Khan A. Wahid

**Affiliations:** Department of Electrical and Computer Engineering, University of Saskatchewan, Saskatoon, SK S7N5A9 Canada

**Keywords:** Color map, Color reproduction, Wireless capsule endoscopy, Endoscopic images, Enhancement, Sharpening

## Abstract

In this paper, we present a real-time preprocessing algorithm for image enhancement for endoscopic images. A novel dictionary based color mapping algorithm is used for reproducing the color information from a theme image. The theme image is selected from a nearby anatomical location. A database of color endoscopy image for different location is prepared for this purpose. The color map is dynamic as its contents change with the change of the theme image. This method is used on low contrast grayscale white light images and raw narrow band images to highlight the vascular and mucosa structures and to colorize the images. It can also be applied to enhance the tone of color images. The statistic visual representation and universal image quality measures show that the proposed method can highlight the mucosa structure compared to other methods. The color similarity has been verified using Delta E color difference, structure similarity index, mean structure similarity index and structure and hue similarity. The color enhancement was measured using color enhancement factor that shows considerable improvements. The proposed algorithm has low and linear time complexity, which results in higher execution speed than other related works.

## Background

Cancer is currently the second-leading cause of death in the United States (Siegel et al. 2015). Furthermore, in 2015 cancer in the digestive system may cause the second highest number of fatalities among all sites (Siegel et al. 2015). Endoscopy plays an important role in diagnostic of colon rectum cancer at an early development stage (Hosokawa et al. 2008). As a result, mortality role for diseases like the stomach cancer, colon cancer, and ulcerative colitis has been drastically decreased in the recent years (Stock et al. 2011). In addition, the ability to capture digital pictures has paved the way for the new field of computer-aided decision support system (CADSS) in medical endoscopy (Liedlgruber and Andreas 2011). These systems focus on aiding in different decision making from endoscopic images such as assessment of different diseases (Kumar et al. 2012; Cong et al. 2014), bleeding detection (Sainju et al. 2014) and frame of interest extraction (Li et al. 2014). In both cases for diagnosis by physician or CADSS, image quality plays a critical role. Wireless Capsule Endoscopy (WCE), as an alternative to the wired endoscopy, offers physicians the capability of examining the interior of the small intestine with a noninvasive procedure (Brownsey and Michalek 2010). In WCE, a battery powered camera is placed in a capsule. When the patient swallows this capsule, it send picture continuously from the gastrointestinal tract (GI). Due to its limited power consumption, the WCE suffers from low image quality (Nakayoshi et al. 2004). Even a high-definition white light endoscopy cannot always detect all mucosal or vascular abnormalities of different positions of GI tract. In both cases for wireless and wired endoscopy, improved image quality can greatly increase early detection and reduce the miss rates of the detection of the mucosal or vascular abnormalities (Liedlgruber and Andreas 2011).

There are both pre-processing and post-processing methods that can significantly enhance mucosal or vascular characteristics in endoscopic images. Pre-processing systems like narrow band imaging (NBI) and auto-fluorescence imaging (AFI) use rotating filters in front of the light source sequentially generating red, blue and green light for tissue illumination (Schmitz-valckenberg et al. 2008; Pohl et al. 2007). Special light sources and filters are utilized to enhance the mucosal structure in the resultant images in NBI and AFI at a cost of higher hardware complexity and power consumption. As an alternative, post-processing system such as virtual chromoendoscopy (CE) decomposes the image into various wavelengths and produces pseudo color added image with enhanced mucosal surface contrast (Chiu et al. 2007). Several researchers concluded that NBI appeared to be a less time-consuming and efficient alternative to CE for the detection of neoplasia. However, NBI has a higher miss rate than CE (Khan and Wahid 2011; Nass and Connolly 2010). Additionally, neither NBI nor CE can improve the adenoma detection or reduce miss rates during screening colonoscopy. As found in works in (Nass and Connolly 2010) and (Khan and Wahid 2011), NBI and CE showed no difference in terms of diagnostic efficacy. Based on the success of CE, several researchers proposed post processing enhancement method for endoscopic image. For example, Okuhata et al. has proposed a real-time enhancement procedure based on retinex theory (Okuhata et al. 2013) and Vogt et al. has proposed real time endoscopic image enhancement scheme based on color normalization (Vogt et al. 2003).

In image processing in general, a well-known procedure for image enhancement is to enhance the luminance channel only while keeping the chrominance channel unchanged (Gonzalez and Woods 2002). Due to psycho-visual redundancy, human eyes are more sensitive to the enhancement of brightness than color. There are a several well-known methods available for enhancing the grayscale image, which can be broadly divided into two categories. Techniques such as contrast stretching (CS) (Wang and Bovik 2002), high boost filtering (HBF) (Srivastava et al. 2009) and unsharp masking (UM) (Polesel et al. 2000) work on the local gradient of the image. On the other hand, techniques such as histogram equalization (HE), contrast limited adaptive histogram equalization (CLAHE) (Zuiderveld 1994) and brightness preserving dynamic fuzzy histogram (BPDFHE) (Sheet et al. 2010) work on the global gradient of the image. The global gradient methods are effective for the low contrast images that contain a single object or no apparent contrast change between object and background (Cheng and Shi 2004).

On the other hand, the psycho-visual redundancy can be utilized to reduce the power consumption in endoscopy. This phenomenon is often utilized in image compression by sending only the grayscale image or the brightness channel. The grayscale image is later colorized using a similar tone theme images, which results in a significant save of power, memory, and bandwidth (Khan et al. 2015). There are several color reproduction algorithms available in the literature. In (Welsh et al. 2002), color information is retrieved from a target swatch. Each pixel in the grayscale image is matched with the pixel in the target swatch based on Euclidean distance metrics. The color is then copied from the matched pixel in the target swatch. This algorithm suffers from high computational complexities and processing time. In another work (Horiuchi and Hirano 2003), the authors have used a set of seed points and their respective color vectors in the RGB format with a YUV-based classification. In (Levin et al. 2004), a quadratic objective function based optimization method is used to interpolate the U and V components of the YUV color space over the entire image using a set of color scribble lines. In (Korostyshevskiy 2006), pseudo colors are employed to colorize the grayscale image using different 64 × 3 color matrices. It does not reproduce a visually appealing color on the entire image and introduces blurriness on the high contrast edges. None of those mentioned above methods has ever been applied to endoscopic imaging. In a recent work by our group, a color enhancement scheme is presented that is dedicated to enhance endoscopic images (Imtiaz et al. 2013 and Imtiaz and Khan 2014). Although this color enhancement scheme is very promising in terms of the color enhancement factor (CEF), it suffers from high algorithm complexities.

In this paper, we propose a dictionary based color reproduction method with low complexities, high color similarity and high CEF. This method enhances the visual quality of GI images. We have also shown three possible scenarios in endoscopy where the proposed method is applicable. The performance of the proposed method is assessed on a relatively diverse dataset based on the reduction of image degradation, structural similarity, and CEF.

## Proposed method

**Fig. 1:**
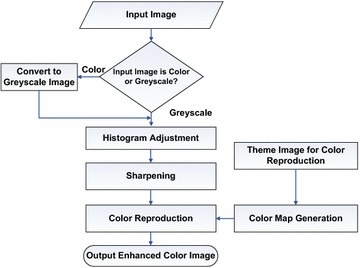
Flowchart of the proposed color image reproduction technique

The proposed method has two steps as shown in Fig. 1. At first, the grayscale images are enhanced by histogram shifting and image sharpening. This results in a high contrast image with better visibility of the mucosa layer. In the second step, the grayscale image is colorized using a theme image, taken from nearby anatomical location, by utilizing a dictionary based color reproduction method.

### Enhancement stage

Endoscopic images have the intrinsic characteristics of low contrast and inhomogeneous brightness, which stems from the random steering motion of the camera and bending and waving nature of the gastric organs (Vogt et al. 2003). The goal in enhancement stage is to increase the contrast and reduce the inhomogeneous brightness.

The enhancement stage works on the grayscale channel of the image. At first, the input color image P is converted to a grayscale image, Y, using Eq. (1):

1 $$ Y = \frac{R}{4} + \frac{G}{2} + \frac{B}{4} $$

Here R, G, and B represent the intensity of red, green and blue channel of the RGB color image respectively using Eq. (1), which uses division by a power of two, so it can be very easily implemented using a shift register. This type of implementation results in a simpler hardware implementation than other conversion methods.

On the other hand, for a gray scale image:

2 $$ Y = P $$

**Fig. 2:**
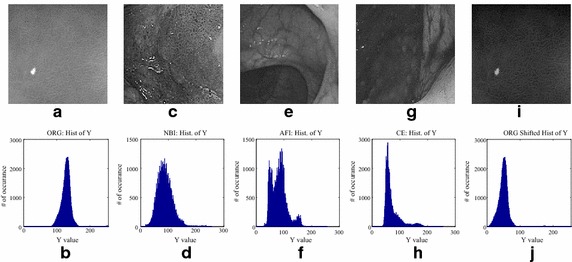
(**a**, **b**) gray-scale endoscopic image and its histogram; (**c**, **d**) NBI image and its histogram; (**e**, **f**) AFI image and its histogram; (**g**, **h**) virtual CE image and its histogram; (**i**, **j**) histogram shifted image of **a** and its new histogram

To reduce the over exposure effect, we have analyzed several images of NBI, AFI, and virtual CE systems and found that the histogram of these images has more content in low regions. The results are shown in Fig. 2. As a result, at this stage of our method, we adjust the histogram by shifting it to the left using Eq (3). Here Ymin is the minimum intensity level in Y.

3 $$ Y^{h} = Y - Y_{ min } $$

This shifting produces a darkening effect that improves the visibility of mucosa layer. All processed images have a dominance of low-intensity pixel compare to the white lighting image (WLI).

The second step in the enhancement stage consist of a modified UM. First the vein or the mucosa layer are extracted from the image using Eq. (4). Here *g* (*Y*^*h*^) indicates the blurred image created by convoluting the image with Gaussian filter.

4 $$ \Delta Y = Y^{h} - g(Y^{h} ) $$

Then the sharpened image is produced by Eq. (5).

5 $$ Y^{EH} = Y^{h} + \lambda \Delta Y $$

**Fig. 3:**
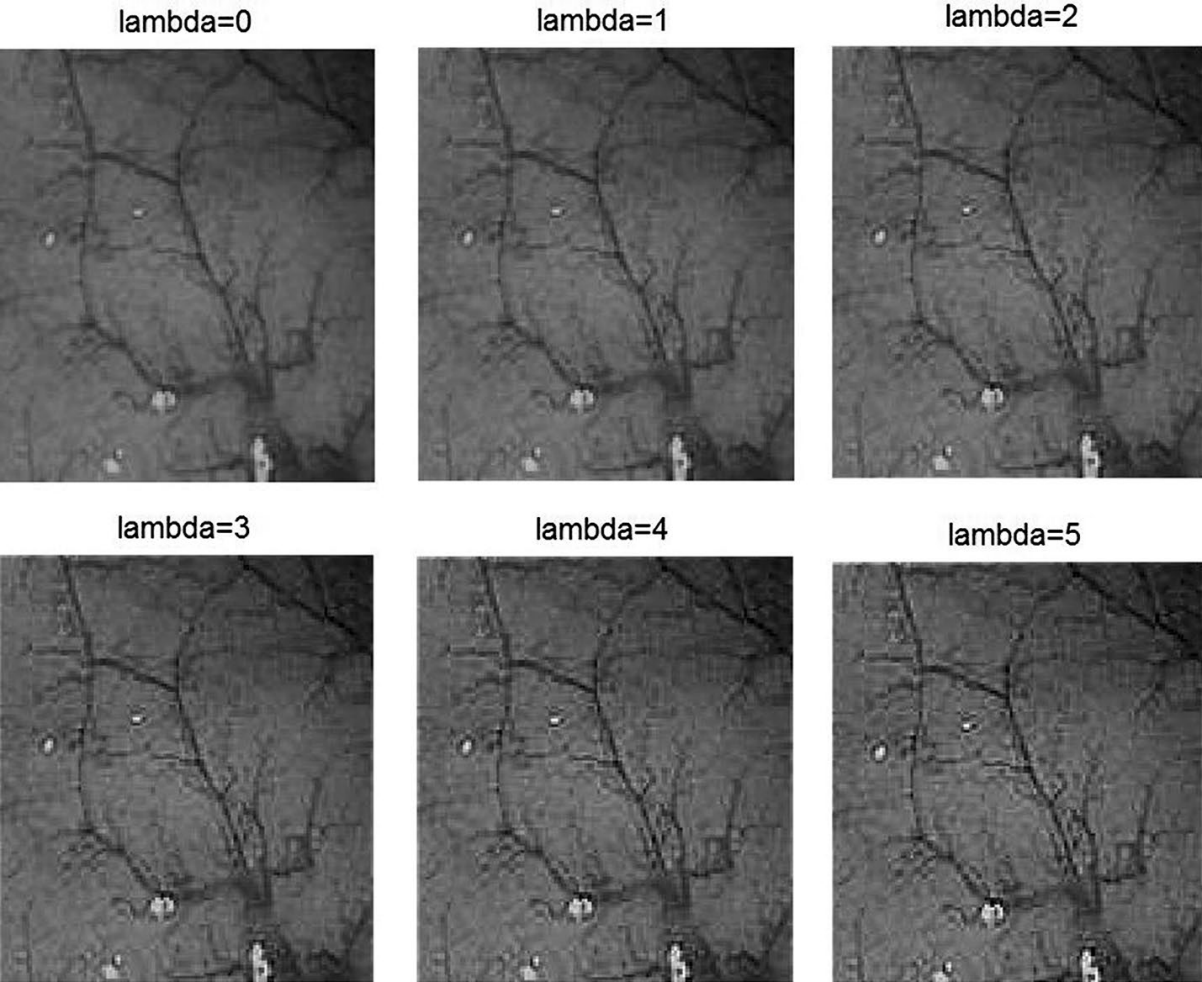
Sharpened image having different value of lambda

Here *λ* is the sharpening factor. Sharpening factor controls the amount of sharpening in the resulting image. Higher the value of sharpening factor, more enhancements can be achieved. But with very high value of λ, the noise presented in the edge image, produced by Eq. (4), gets magnified. As a result, image artifacts and ringing effect would be introduced in the resultant image. In Fig. 3, images sharpened using different sharpening factor, λ, are shown. Here, we can observe that the increment of sharpening factor is eventually increasing the visibility of mucosa structure in the gray endoscopic image. The specific sharpening factor for each figure is given with the figure.

From the experiment it has been found the values of sharpening factor in the range 2–8 provide the best result.

### Proposed color reproduction algorithm

At this stage, color is added to the generated enhanced grayscale image to produce a colorized image. The color information is retrieved using the available color image of the nearby anatomical location. Then using the color similarity between the theme image and the grayscale image, the algorithm reproduces the color of the enhanced grayscale image. We have prepared a database of color WCE pictures taken from (Gastrolab—the gastrointestinal site 1996; Atlas of gastrointestinal endoscopy 1996) for different locations of the GI tract from where a theme image is chosen manually. This part of the algorithm consists of two steps. color map generation and color reproduction.

#### Color map generation

The color map, Z, is a lookup table that maps luminance components to corresponding color components, providing the R, G, B value for a pixel with a particular value of luminance Y. The generated color map is dynamic in nature as they change when a new theme image is chosen. Color map is expressed as below:

6 $$ Z:Y \to \{ R,G,B\} $$

First a color map is generated from a theme image T. Each color pixel is converted to luminance, Y using Eq. (1). Then the corresponding R, G and B values are listed in a color map lookup table. Since there is no one to one correspondence between Y and R, G, B, there may be multiple combinations of R, G, B with a same value of Y. In these cases, the mean values of R, G, B are taken. For an example, RGB triplet (32, 16, 8) and (0, 28, 0) both will produce the same Y value 14. In this case, in the lookup table the average of these combinations, that is (16, 22, 4), will be saved for Y = 14. These may produce color artifacts in the colorized image. However, since, for an anatomically nearby location image, the correlation between the color and luminance would be very high, the trend between the luminance and color of the theme image would provide sufficient information about the color for the enhanced grayscale image.

After completing the table, there might be some empty slots in the color map table if all *Y* values from 0 to 255 are not generated from the image pixels. The empty slots are filled by linear interpolation between two nearest neighboring entries.

For instance, if there are some consecutive empty slots from row *M* to row *M* + *d* in the lookup table, the empty slots entries are calculated using Eq. (7), where *X* represents *R*, *G*, *B* and *i* range from *0* to *d*.

7 $$ X_{M +\,i} = X_{M - 1} + \frac{{X_{M - 1} - X_{M +\, d + 1} }}{d + 2} \times \left( {i + 1} \right) $$

**Fig. 4:**
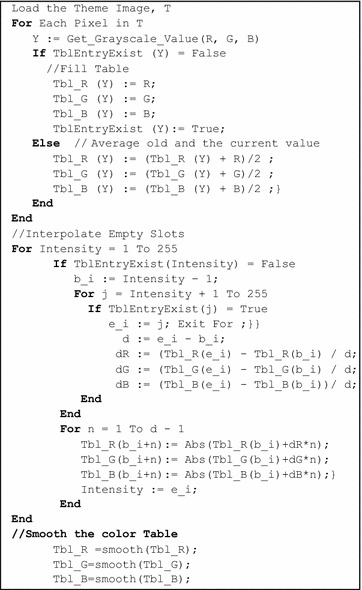
Pseudo code for color map generation

**Fig. 5:**
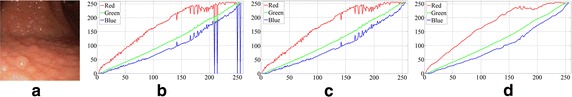
Color map generation procedure, **a** a theme endoscopy image (T) at gastric corpus location; **b** plot of the color map without interpolation; **c** plot of the color map with interpolation; **d** plot of the color map with interpolation and smoothing

The first and last entry of the table is kept fixed presenting full black (0, 0, 0) and full white (255, 255, 255) namely. A pseudo code for color map generation is shown in Fig. 4. Figure 5 shows a theme image and the corresponding plot of the color map generated using the proposed scheme. After interpolation, the generated color map shows a lot of ripple which can introduce color artifacts later in the colorized image. To remove this ripple, a smoothing function is applied to the generated color map.

The optimum smoothing function was determined by calculating the difference between the original grayscale image and grayscale image regenerated from the colorized image using the generated color map. Table 1 shows the result. From the result, we conclude that linear local regression with span 19 provides the best outcome in this case. In the future in this paper, wherever it is not specified, linear local regression with Span 19 was used.

**Table 1:** PSNR measures (in dB) with different smoothing function applied to the color table

Span	5	9	15	19
Smoothing function				
Moving average	51.07	51.42	51.50	51.16
Linear local regression	51.27	51.18	51.14	52.06
Quadratic local regression	51.11	51.94	51.50	51.22
Savitzky–Golay	50.46	51.16	51.22	50.91
Robust linear local regression	51.26	49.29	49.43	49.53
Robust quadratic local regression	48.82	49.85	49.40	49.16

#### Applying the color map

After the new color map has been generated, the enhanced grayscale image is colorized using the lookup table. For each pixel, the R, G and B values are looked up from Z, using the luminance value of that pixel. For example, if a pixel has a luminance value of 100, the corresponding R, G and B value in the lookup table would be the color value for that pixel. In this way, the output colored image, O, is produced.

8 $$ Z:\forall Y \in EH \to \left\{ {R,G,B\} } \right. = O $$

## Experimental results

**Fig. 6:**
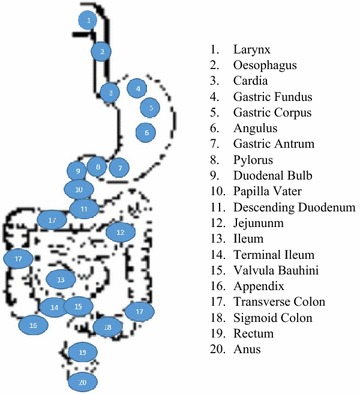
Position of GI track

This section illustrates possible applications where the proposed method is applied. They are divided into three categories and discussed below. For these experiments, several images and videos were collected from the GastroLab (Gastrolab—the gastrointestinal site 1996; Atlas of gastrointestinal endoscopy 1996) database. In addition, we have prepared and used a database of 100 theme images taken from 20 different anatomical locations of human GI tract (See Fig. 6). The theme images were taken manually from nearby anatomical location for the experiments.

### Category 1: low contrast WLI images

**Fig. 7:**
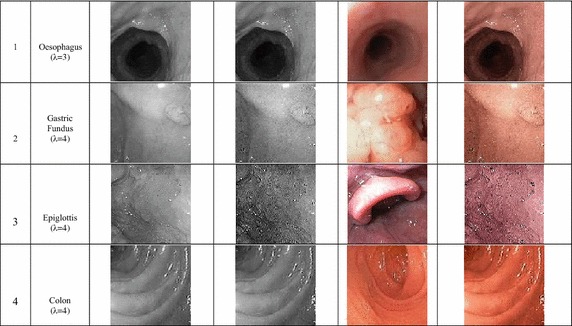
Category 1: preprocessing and color reproduction of grayscale WLI images

Firstly, we have used several WLI grayscale image whose color information was absent. WLI images generally suffer from low contrast as evident from the input original images in Fig. 7. The proposed algorithm can improve the visual quality of the system by enhancing the mucosa layer and retrieving color information from a theme image taken from a nearby anatomical location. The results are shown in Fig. 7. It can be seen that, although the input original images and the corresponding theme images have a different structure, the reproduced color images have a very similar color tone to the theme image. On the other hand, the sharpening introduced in the preprocessing stage increases the contrast and make the subtle features more visible (compare the input original image with preprocessed grayscale image). Two conclusion can be drawn from this experiment. Firstly, the proposed method can be used for colorizing and enhancing grayscale WLI image for which color information is unavailable. Secondly, since color information is not required, it can be omitted from transmission of WLI image, resulting in a more simple capture device, lower transmission power and lower bandwidth requirement. The grayscale image can be later colorized using the proposed method and the database of theme image for different GI location.

### Category 2: raw NBI images

**Fig. 8:**
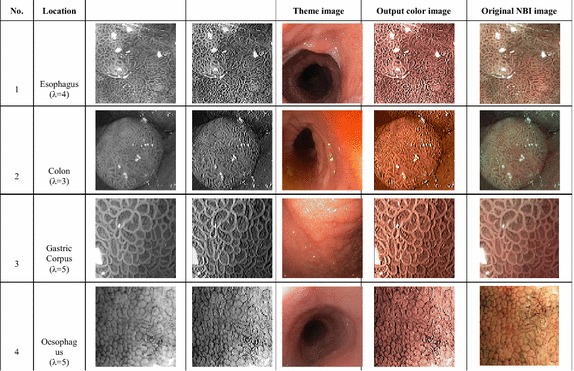
Category2: preprocessing and color reproduction of grayscale NBI images

In NBI images, special lighting is utilized to enhance the mucosa layer. Lights of 415 and 540 nm wavelengths are used to illuminate the mucosa surface; the reflected light from the mucosa is captured in a monochromic CCD image sensor (Dung and Wu 2010). The grayscale images from the CCD image sensor are then passed to an image processor where pseudo color is added to the images (Evis Lucera Spectrum family brochure (online) 2014). The proposed method can be utilized in two ways. It can directly colorize the raw NBI image and also provide greater enhancement. On the other hand, it may replace the color transformation system by colorizing the NBI image using the WLI image as the theme image. We used the method to colorize several raw NBI images, and the results are shown in Fig. 8. It can be seen from the figure that the output images have better visibility of the mucosa layer than the input raw NBI images. If we compare the corresponding original NBI image, the structures are better defined in our method due to image preprocessing provided by the proposed method.

### Category 3: tone enhancement

**Fig. 9:**
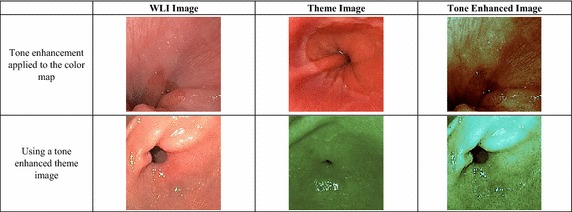
Category 3: tone enhanced color reproduction

In CE systems, such as i-scan technology, it has been shown that mapping tone of a WLI image into a different color map can improve the visibility of the lesion and mucosal layer (Kodashima and Fujishiro 2010). In this experiment, we want to demonstrate the flexibility of the proposed method in providing similar type of tone enhancement. Firstly, tone mapping model can be applied directly to the color map produced from the theme image. For example, TE-g (Kodashima and Fujishiro 2010) tone mapping is applied to the color map of the theme image shown in Fig. 9. The resultant color map is then used for colorizing the WLI image, and a tone enhanced image similar to image produced by i-scan TE-g is produced. By comparison to the WLI image, tone enhanced image has more crisp mucosa structure due to image sharpening in the preprocessing step. The tone enhancement further improve the visibility of the mucosa structure.

In the second case, a tone enhanced endoscopic image can be used as a theme image to transfer its characteristics to the WLI images. For example, in the second image in Fig. 9, a tone enhanced theme image from a nearby anatomical location is used to reproduce the color of the original WLI image. The output image showed more enhanced mucosal structure than the original image. In both cases, we have applied the TE-g tone enhancement proposed by i-scan (Kodashima and Fujishiro 2010).

## Performance analysis

In this section, experiments are conducted to prove the validity of the proposed method in the enhancement of the mucosal structure and color reproduction. Altogether 178 images with different physiological characteristics, taken from GastroLab (Gastrolab—the gastrointestinal site 1996) and Atlas (Atlas of gastrointestinal endoscopy 1996) databases, were used for the purpose of comparison. For theme images, we have used the database of 100 WLI images taken from 20 different location in GI tract. There was no overlapping between these two database meaning that the images used for experimentation were different than the images used for color theme image. The sharpening factor used for different pictures are specified in the figure. Both objective and the subjective evaluation were considered as performance metrics. The objective method evaluated the enhancement, quality of the images and similarity between the original and colorized images. On the other hand, the quality of the proposed method in mucosal structure enhancement has been verified visually by conducting a survey among gastroenterologists.

### Reducing the effect of over lighting and low contrast

Image in capsule endoscopy suffers from inhomogeneous lighting and low contrast. In a recent study, Sdiri et al. has shown that the contrast enhancement improved the stereo matching performance and classification results (Sdiri et al. 2015). To measure the performance of the proposed method in contrast enhancement, Statistics of visual representation (SVR) (Balas et al. 2009; Jawahar and Ray 1996) was used. SVR compares the contrast and intensity of the original image and enhanced image. High contrast measurement means that the resultant image has higher contrast than the original image. Similarly, high intensity measurement indicates that the processed image has a higher average intensity than the original image. In addition, to evaluate the image quality, Universal Image Quality (UIQ) (Wang and Bovik 2002) was used. UIQ is a mathematically defined parameter that evaluates the image quality on three factors: loss of correlation, luminance distortion and contrast distortion. UIQ value closer to +1 indicates good quality while value closer to −1 indicates bad quality.

The proposed method was compared with other enhancement methods in terms of SVR and UIQ in Table 2. Here the average value was shown for a database of 178 images. Table 2 shows that the proposed method can increase the overall contrast level in an image compared to other methods, but at the same time can decrease the overall intensity level. Thus, statistically it can be stated that this method will provide an image with higher contrast and darker tone. The result also showed that the proposed method provides the highest quality image in terms of UIQ value.

**Table 2:** SVR measures with other related works

	Contrast measurement	Intensity measurement	Universal image quality index
Proposed method (178 images)	1.3711	-0.22901	0.9241
Adaptive histogram equalization (AHE)	0.2393	0.08304	0.5722
Contrast stretching (CS)	0.7022	0.14686	0.9244
High boost filtering (HBF)	−0.0033	0.00049	0.2633
Unsharp masking (UM)	0.03975	0.00002	0.9528

**Fig. 10:**
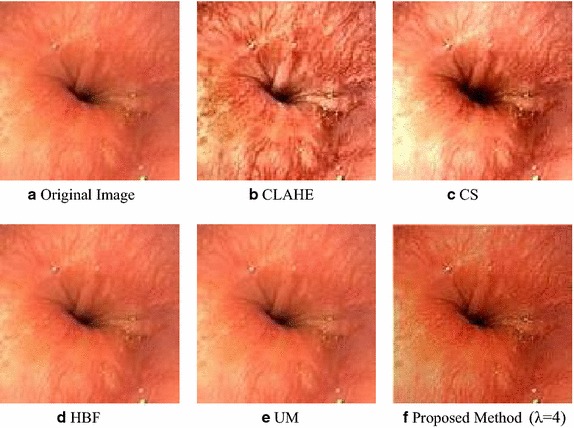
Visual representation of color enhancement of different algorithms

In Fig. 10, a visual comparison of the algorithms is shown for a sample image taken from the database. As evident from the figure, the proposed method increases the contrast level as well as sharpens the subtle details.

### Color similarity test and color enhancement factor (CEF)

In this section, the proposed color reproduction is evaluated in terms of color similarity and color enhancement. Color similarity takes into account the structural similarity between the original image and colorized image. In this paper, we have used CIE94 delta-E color difference (Robertson 1990), structure similarity index (SSIM), mean structure similarity index (MSSIM) (Wang et al. 2004) and structure and hue similarity (SHSIM) (Shi et al. 2009) for evaluating color similarity. All these parameters provide the difference in color between the original and processed image. CIE94 measures the color differences between processed and original image in LAB color space. In CIE94, value close to 2.3 indicates that the color difference between two images is the lowest. SSIM, MSSIM and SHSIM measure the color similarity in the chrominance planes in YCbCr color space. The indices of these parameters close to 1 indicate higher similarities between two structures and chrominance planes. The results are compared with other color reproduction methods and presented in Table 3. We can see that the average SSIM, MSSIM and SHSIM indices are higher than others, with a color difference close to 2.3. All these values indicate that the colorized frames are very close to the original images in terms of structural similarities.

**Table 3:** Color similarity and enhancement assessment

	CIE94	SSIM	MSSIM	SHSIM	CEF
Proposed (178 images)	2.36	0.9996	0.9792	0.9987	1.3322
Welsh et al.	4.1	0.9001	0.8605	0.8578	0.7615
Korostyshevskiy	0.6	0.8907	0.8714	0.8001	0.8853
Imtiaz et al.	3.01	0.9945	0.9967	0.9942	1.1520
Okuhata et al.	1.18	0.9922	0.7479	0.9603	0.6840
Vogt et al.	4.79	0.9847	0.4078	0.2826	1.0429

**Fig. 11:**
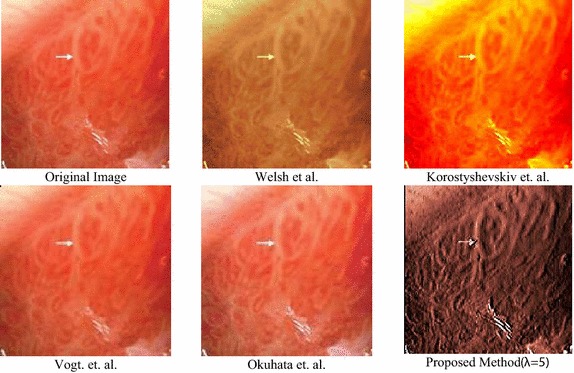
Visual representation of color enhancement of different color reproduction algorithms

The proposed method was also evaluated in terms of the CEF. A no-reference performance metrics called colorfulness metric (CM) was used for this purpose (Susstrunk and Winkler 2003). In this paper, we have used the ratios of CMs between the processed and original image for observing the CEF. The higher the CEF value, the higher the high-frequency components and lesser the color artifacts. Table 3 shows that our method provides the highest CEF while keeping a strong structural similarity. The color enhancement is further evident in the visual comparison of a sample image in Fig. 11.

### Algorithm complexity

The proposed method has an extremely low complexity that results in very fast execution time. The time required to generate an enhanced color image from the original image for different image sizes using various methods are shown in Table 4. All the algorithms were implemented in MATLAB 2013 platform and the experiments were conducted on a PC having Intel (R) Core (TM) i3-2310M CPU @ 2.10 GHz and 6 GB of RAM. Here, it is noticeable that the proposed method is faster than all other methods except Korostyshevskiv’s algorithm. Although the execution time of Korostyshevskiv’s algorithm (Korostyshevskiy 2006) is lower than ours, the quality of the color reproduction is worse as shown in Fig. 11 and Table 3.

**Table 4:** Algorithm complexity assessment

Methodology	Image size	Image enhance. time (sec)	Color reprod. time (sec)	Total time (sec)
Proposed (178 images)	256 × 256	0.06	1.50	1.56
	512 × 512	0.21	6.31	6.52
Imtiaz et al.	256 × 256	0.39	28.25	28.64
	512 × 512	1.52	113.31	114.83
Welsh et al.	256 × 256	–	64.46	64.46
	512 × 512	–	235.14	235.14
Korostyshevskiy	256 × 256	–	0.19	0.19
	512 × 512	–	0.47	0.47
Okuhata et al.	256 × 256	0.21	–	0.21
	512 × 512	1.37	–	1.37
Vogt et al.	256 × 256	0.11	–	0.11
	512 × 512	0.56	–	0.56

### Subjective evaluation by gastroenterologists

To perform a preliminary subjective evaluation, we have conducted a primitive survey among several professional gastroenterologists. In the survey, three original and processed image pairs were shown to several gastroenterologists and their opinion was requested on whether they agree with the following statement: “the enhanced image can provide better visibility of the mucosa surface than the original image”. Here, the original image indicates the raw color endoscopic image and enhanced image indicates the processed image using the proposed method. The results of the survey are summarized in Table 5. The average mean opinion score (MOS) (Viera and Garrett 2005) for all images is 1.17, which is interpreted as “agree”. The result indicates that the proposed method can highlight the mucosa structures.

**Table 5:** Survey results

Image #	Doctor 1 S_1_	Doctor 2 S_2_	Doctor 3 S_3_	Doctor 4 S_4_	MOS
1	1	−1	1	1	0.50
2	1	2	1	2	1.50
3	1	2	1	2	1.50
Average mean opinion score (MOS)					1.17

Strongly agree = 2, agree = 1, neutral = 0, disagree = −1, strongly disagree = −2; MOS = sum (S_1_:S_4_)/4

## Conclusions

This paper presents a novel dictionary based algorithm for colorizing and enhancing a grayscale endoscopic image. The color information and the enhancing improve the visual quality of the endoscopic image. The proposed algorithm generates a dynamic color map from a color theme image. Then the color map is applied to the enhanced grayscale image to produce a color image having a similar color tone of the theme image. The quality of the generated enhanced color image is evaluated using several standard performance metrics, which showed better performance compared to many existing methods. This method can be used in enhancing and colorizing low contrast grayscale WLI images and as an alternative to the color transformation system for NBI system.
